# Sexual Intercourse Among High School Students — 29 States and United States Overall, 2005–2015

**DOI:** 10.15585/mmwr.mm665152a1

**Published:** 2018-01-05

**Authors:** Kathleen A. Ethier, Laura Kann, Timothy McManus

**Affiliations:** 1 Division of Adolescent and School Health, National Center for HIV/AIDS, Viral Hepatitis, STD and TB Prevention, CDC.

Early initiation of sexual activity is associated with having more sexual partners, not using condoms, sexually transmitted infection (STI), and pregnancy during adolescence (*1*,*2*). The majority of adolescents initiate sexual activity during high school, and the proportion of high school students who have ever had sexual intercourse increases by grade; black students are more likely to have ever had sexual intercourse than are white students (*3*). The proportion of high school students overall who had ever had sexual intercourse did not change significantly during 1995–2005 (53.1% to 46.8%) (Division of Adolescent and School Health, National Center for HIV/AIDS, Viral Hepatitis, STD, and TB Prevention, CDC, unpublished data). To assess whether changes have occurred in recent years in the proportion of high school students who have ever had sexual intercourse, CDC examined trends overall and by grade, race/ethnicity, and sex among U.S. high school students, using data from the 2005–2015 national Youth Risk Behavior Surveys (YRBSs) and data from 29 states* that conduct the YRBS and have weighted data. Nationwide, the proportion of high school students who had ever had sexual intercourse decreased significantly overall and among 9th and 10th grade students, non-Hispanic black (black) students in all grades, and Hispanic students in three grades. A similar pattern by grade was observed in nearly half the states (14), where the prevalence of ever having had sexual intercourse decreased only in 9th grade or only in 9th and 10th grades; nearly all other states saw decreases in some or all grades. The overall decrease in the prevalence of ever having had sexual intercourse during 2005–2015 is a positive change in sexual risk among adolescents (i.e., behaviors that place them at risk for human immunodeficiency virus, STI, or pregnancy) in the United States, an overall decrease that did not occur during the preceding 10 years. Further, decreases by grade and race/ethnicity represent positive changes among groups of students who have been determined in previous studies to be at higher risk for negative outcomes associated with early sexual initiation, such as greater numbers of partners, condom non-use, teen pregnancy, and STI (*1*–*3*). More work is needed to understand the reasons for these decreases and to ensure that they continue.

The national YRBS is a biennial, school-based survey of U.S. high school students conducted by CDC. For each survey, a three-stage cluster sample design was used to produce a nationally representative sample of students in grades 9–12 who attend public and private schools. During 2005–2015, sample sizes ranged from 13,917 to 16,410, and overall response rates ranged from 60% to 71%. Data were weighted to yield nationally representative estimates.

Data from 29 state YRBSs conducted by state health and education agencies also were included in this report. In each state survey, a two-stage cluster sample design was used to produce representative samples of public school students in 28 states and in public and private school students in one state. During 2015, sample sizes across state surveys ranged from 1,313 to 14,837; overall response rates ranged from 60% to 81%. Data were weighted to yield representative estimates by state.

Survey procedures for the national and state surveys were designed to protect students’ privacy by allowing anonymous and voluntary participation. Local parental permission procedures were followed before survey administration. Students completed the self-administered questionnaire during one class period and recorded their responses directly on a computer-scannable booklet or answer sheet. Each questionnaire included the following question to ascertain prevalence of ever having had sexual intercourse: “Have you ever had sexual intercourse?” Response options were “yes” and “no.” No definition for sexual intercourse was provided.

For the national YRBS, prevalence estimates were computed overall and by grade (9th, 10th, 11th, or 12th), sex (male or female), and race/ethnicity (non-Hispanic white [white], black, or Hispanic). For the state YRBSs, prevalence estimates were computed by grade. Statistical software was used to account for the complex sample designs during analyses.

Logistic regression analyses were used to account for all available estimates; control for changes in sex, grade, and race/ethnicity over time; and assess statistically significant (p<0.05) long-term linear and quadratic trends in ever having had sexual intercourse during 2005–2015. A quadratic trend indicates a significant but nonlinear trend in prevalence over time. Both a linear and quadratic trend are possible because the linear trend indicates the direction of the trend from the start to the end of the time frame, and the quadratic trend indicates a nonlinear change within the time frame. For the national YRBS, race/ethnicity data are presented for black, white, and Hispanic students only.

Nationwide, during 2005–2015, a significant linear decrease in the prevalence of ever having had sexual intercourse among all students in grades 9–12 (46.8% to 41.2%) was identified (Table) (Figure 1). A significant linear decrease also was identified among male (47.9% to 43.2%), female (45.7% to 39.2%), black (67.6% to 48.5%), and Hispanic (51.0% to 42.5%) students. Among black students, a significant quadratic trend also was identified. The prevalence of ever having had sexual intercourse among black students did not change between 2005 (67.6%) and 2009 (65.2%), but subsequently decreased from 2009 (65.2%) to 2015 (48.5%).

**TABLE:** Trends in prevalence of ever having had sexual intercourse among high school students, by sex, race/ethnicity, and grade in school — National Youth Risk Behavior Surveys, United States, 2005–2015

Characteristic	Prevalence, %						Trend p-value*	
	2005	2007	2009	2011	2013	2015	Linear	Quadratic
**Total**	**46.8**	**47.8**	**46.0**	**47.4**	**46.8**	**41.2**	**0.0069^†^**	**0.0770**
**Sex**								
Male	47.9	49.8	46.1	49.2	47.5	43.2	0.0106^†^	0.1919
Female	45.7	45.9	45.7	45.6	46.0	39.2	0.0176^†^	0.0648
**Race/Ethnicity**								
White^§^	43.0	43.7	42.0	44.3	43.7	39.9	0.3711	0.4370
Black^§^	67.6	66.5	65.2	60.0	60.6	48.5	0.0000^†^	0.0163^†^
Hispanic	51.0	52.0	49.1	48.6	49.2	42.5	0.0003^†^	0.1194
**9th grade**	34.3	32.8	31.6	32.9	30.0	24.1	0.0000^†^	0.0541
**Sex**								
Male	39.3	38.1	33.6	37.8	32.0	27.3	0.0000^†^	0.1789
Female	29.3	27.4	29.3	27.8	28.1	20.7	0.0080^†^	0.0713
**Race/Ethnicity**								
White^§^	29.4	25.8	24.9	27.3	26.5	21.3	0.0614	0.8057
Black^§^	55.4	52.5	51.5	48.2	43.1	31.4	0.0000^†^	0.0417^†^
Hispanic	40.5	39.7	37.9	36.8	31.6	25.9	0.0001^†^	0.0637
**10th grade**	42.8	43.8	40.9	43.8	41.4	35.7	0.0449^†^	0.1769
**Sex**								
Male	41.5	45.6	41.9	44.5	41.1	37.9	0.1283	0.2272
Female	44.0	41.9	39.6	43.0	41.7	33.5	0.0506	0.2927
**Race/Ethnicity**								
White^§^	37.5	38.1	34.7	38.4	35.4	32.8	0.3625	0.7079
Black^§^	66.4	66.4	64.8	58.4	62.6	47.3	0.0002^†^	0.0784
Hispanic	46.9	49.1	44.8	46.5	45.8	36.0	0.0095^†^	0.0674
**11th grade**	51.4	55.5	53.0	53.2	54.1	49.6	0.3631	0.1934
**Sex**								
Male	50.6	57.3	53.4	54.5	54.3	51.2	0.5238	0.1321
Female	52.1	53.6	52.5	51.9	53.9	48.2	0.3724	0.3940
**Race/Ethnicity**								
White^§^	47.3	52.3	49.8	50.5	53.0	47.8	0.7905	0.3021
Black^§^	74.8	74.1	71.3	63.6	63.5	57.2	0.0000^†^	0.8166
Hispanic	55.0	58.1	56.2	56.0	56.7	52.2	0.2288	0.2815
**12th grade**	63.1	64.6	62.3	63.1	64.1	58.1	0.0811	0.2155
**Sex**								
Male	63.8	62.8	59.6	62.6	65.4	59.0	0.3548	0.9941
Female	62.4	66.2	65.0	63.6	62.8	57.2	0.0328^†^	0.0276^†^
**Race/Ethnicity**								
White^§^	60.5	62.1	60.6	62.5	61.0	58.8	0.6164	0.3767
Black^§^	80.0	81.8	79.7	73.9	77.4	63.3	0.0002^†^	0.1352
Hispanic	69.7	70.5	64.7	60.0	69.3	60.7	0.0336^†^	0.5242

* Based on linear and quadratic trend analyses using logistic regression models controlling for grade, sex, and race/ethnicity.

^†^ Statistically significant trend (p<0.05).

^§^ Non-Hispanic; Hispanic persons could be of any race.

**FIGURE 1:**
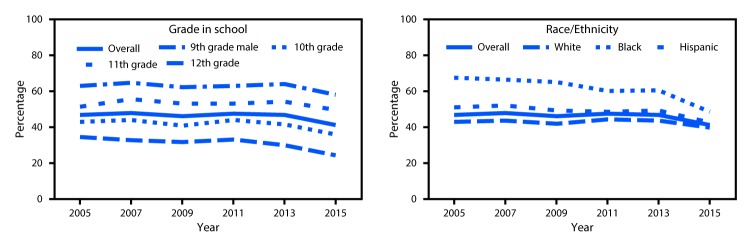
Trends in prevalence of ever having had sexual intercourse among high school students, by grade in school and race/ethnicity — national Youth Risk Behavior Surveys, United States, 2005–2015

During 2005–2015, among 9th grade students, a significant linear decrease in the prevalence of ever having had sexual intercourse was identified overall (34.3% to 24.1%) and among male (39.3% to 27.3%), female (29.3% to 20.7%), black (55.4% to 31.4%), and Hispanic (40.5% to 25.9%) students. Among 9th grade black students, a significant quadratic trend also was identified; prevalence decreased between 2005 (55.4%) and 2011 (48.2%) and then decreased even more sharply from 2011 (48.2%) to 2015 (31.4%). Among 10th grade students, a significant linear decrease in prevalence was identified overall (42.8% to 35.7%) and among black (66.4% to 47.3%) and Hispanic (46.9% to 36.0%) students. Among 11th grade students, a significant linear decrease in prevalence was identified only among black students (74.8% to 57.2%). Among 12th grade students, a significant linear decrease in prevalence was identified among female (62.4% to 57.2%), black (80.0% to 63.3%), and Hispanic (69.7% to 60.7%) students; among 12th grade female students, a significant quadratic trend also was identified. The prevalence of ever having had sexual intercourse did not change between 2005 (62.4%) and 2009 (65.0%) and then decreased from 2009 (65.0%) to 2015 (57.2%). The prevalence of ever having sexual intercourse among white students did not change overall or in any grade.

Across 29 states, a significant linear decrease in the prevalence of ever having had sexual intercourse was identified among only 9th grade students in five states; among only 9th and 10th grade students in nine states; among only 9th, 10th, and 11th grade students in seven states; among 9th, 10th, 11th, and 12th grade students in three states; and among other combinations of grades in three states (Figure 2). In two states (North Dakota and Wyoming), the prevalence of ever having had sexual intercourse did not decrease over time in any grade.

**FIGURE 2:**
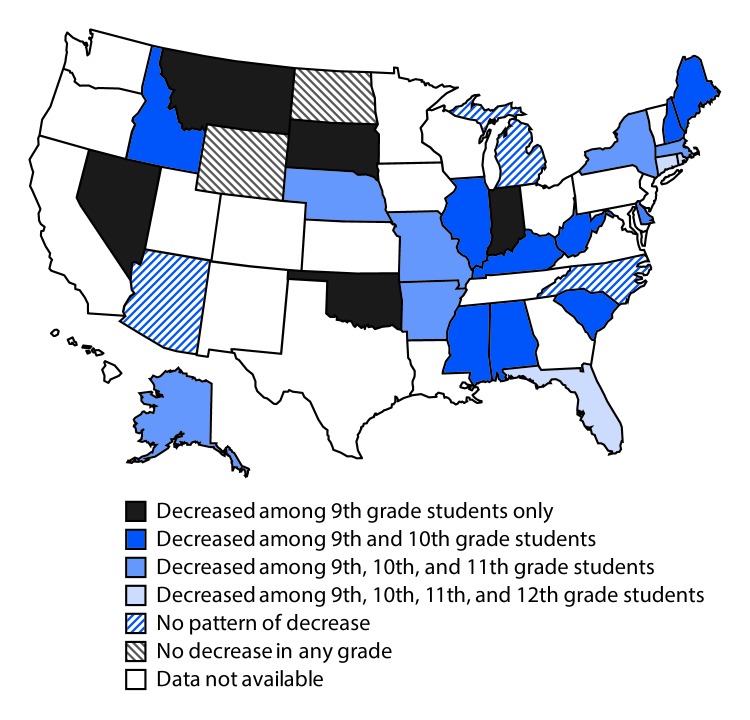
Trends in prevalence of ever having had sexual intercourse among high school students, by grade within state —Youth Risk Behavior Surveys, 29 States, 2005–2015

## Discussion

Nationwide, although the prevalence of ever having had sexual intercourse decreased overall during 2005–2015, closer examination of the data indicated several distinctions by sex, grade, and race/ethnicity. First, among students overall, significant linear decreases were observed among all sex and race/ethnicity subgroups except white students. Second, decreases were seen among 9th and 10th grade students, but not 11th and 12th grade students. A similar pattern was observed in almost half (14) of the states where the prevalence of ever having had sexual intercourse decreased only in 9th grade or only in 9th and 10th grades, and only two states experienced no decreases by grade. Finally, nationwide decreases were seen among black students in all grades and Hispanic students in three grades (9th, 10th, and 12th grades), but no statistically significant decreases were observed among white students in any grade. Thus, these data indicate that during 2005–2015, significant decreases in the percentage of high school students who had sexual intercourse (particularly students in grades 9 and 10 and black students) occurred at the national level and in many states for which data were available. Although these findings cannot be connected directly to any specific intervention, the results indicate that decreases in prevalence of sexual intercourse occurred among the nation’s high school students. During 2005–2015, the United States experienced significant shifts in various influences that might have affected these findings, including changes in technology and the use of social media by youth, requirements and funding for education, and innovations in and federal resources for human immunodeficiency virus infection, STI, and teen pregnancy prevention (*4*,*5*).

The findings in this report are subject to at least two limitations. First, these data apply only to youths who attend school and, therefore, are not representative of all persons in this age group. Nationwide, in 2012, among persons aged 16–17 years, approximately 3% were not enrolled in a high school program and had not completed high school (*6*). Second, the extent of underreporting or overreporting of behaviors cannot be determined, although the survey questions demonstrate good test-retest reliability (*7*).

The decreases in sexual intercourse by grade suggest that fewer students are having sexual intercourse during the earlier years of high school; this finding is especially encouraging. This finding, coupled with decreases in the prevalence of sexual intercourse among black and Hispanic students, represent positive changes among groups of students (e.g., students who have sex at younger ages and black youths) who have been indicated in previous studies to be at higher risk for negative outcomes associated with early sexual initiation, such as higher numbers of partners, non-use of condoms, teen pregnancy, and sexually transmitted diseases. Adolescence is characterized by profound intellectual, emotional, and psychological growth (*8*), all of which could be influenced by sociocultural and educational changes. More research is necessary to understand the contributing factors and the implications of these findings and to examine the contribution of these declines to declines in teenage childbearing and the potential relationship with STI.

**Summary**

**What is already known about this topic?**

Early initiation of sexual activity is associated with more sexual partners, not using condoms, teen pregnancy, and sexually transmitted infection (STI) during adolescence. Most adolescents initiate sexual activity during high school. The percentage of students who had ever had sexual intercourse did not change significantly during 1995–2005 (53.1% to 46.8%).

**What is added by this report?**

Analysis of data from national Youth Risk Behavior Surveys indicated that the proportion of high school students nationwide who had ever had sexual intercourse decreased significantly during 2005–2015 overall, among 9th and 10th grade students, among black students across all grades, and among Hispanic students in three grades. A similar pattern by grade was observed in nearly half of the states with available data.

**What are the implications for public health practice?**

During 2005–2015, the overall decrease in the prevalence of ever having had sexual intercourse is a positive change in the level of sexual risk among adolescents in the United States. The decreases by grade suggest that fewer students are having sexual intercourse during the earlier years of high school. This observation, as well as decreases in the prevalence of sexual intercourse among black and Hispanic students, represent positive changes among groups of students who have been determined in previous studies to be at higher risk for negative outcomes associated with early sexual initiation. Understanding the underlying causes of these decreases in the prevalence of ever having had sexual intercourse can inform strategies to ensure that such decreases continue.

## Footnotes

* Alabama, Alaska, Arizona, Arkansas, Connecticut, Delaware, Florida, Idaho, Illinois, Indiana, Kentucky, Maine, Massachusetts, Michigan, Mississippi, Missouri, Montana, Nebraska, Nevada, New Hampshire, New York, North Carolina, North Dakota, Oklahoma, Rhode Island, South Carolina, South Dakota, West Virginia, and Wyoming.
